# Connecting individual to collective cell migration

**DOI:** 10.1038/s41598-017-10069-8

**Published:** 2017-08-29

**Authors:** Mishel George, Francesco Bullo, Otger Campàs

**Affiliations:** 1 Department of Mechanical Engineering, University of California, Santa Barbara, California USA; 2 California NanoSystems Institute, University of California, Santa Barbara, California USA; 3 Center for Bioengineering, University of California, Santa Barbara, California USA; 4 Department of Molecular, Cell and Developmental Biology, University of California, Santa Barbara, California USA

## Abstract

Collective cell migration plays a pivotal role in the formation of organs, tissue regeneration, wound healing and many disease processes, including cancer. Despite the considerable existing knowledge on the molecular control of cell movements, it is unclear how the different observed modes of collective migration, especially for small groups of cells, emerge from the known behaviors of individual cells. Here we derive a physical description of collective cellular movements from first principles, while accounting for known phenomenological cell behaviors, such as contact inhibition of locomotion and force-induced cell repolarization. We show that this theoretical description successfully describes the motion of groups of cells of arbitrary numbers, connecting single cell behaviors and parameters (e.g., adhesion and traction forces) to the collective migration of small groups of cells and the expansion of large cell colonies. Specifically, using a common framework, we explain how cells characterized by contact inhibition of locomotion can display coherent collective behavior when in groups, even in the absence of biochemical signaling. We find an optimal group size leading to maximal group persistence and show that cell proliferation prevents the buildup of intercellular forces within cell colonies, enabling their expansion.

## Introduction

From embryonic development to tissue regeneration and wound healing, many processes of tissue (re)organization involve the coordinated migration of cells^1^. While some large scale migration processes involve the movements of hundreds of cells (e.g., neural crest cell migration^2^), many migratory events in developmental and disease processes involve small groups (~5–50) of cells^1, 3^, including border cell migration^4^ or lateral line formation^5^. Importantly, there is increasing evidence that cancer invasion and metastases rely on the migration of small clusters of cells rather than individual cells^6^. Despite the existing amount of information regarding the different migratory processes and their molecular control^7–9^, it is unclear how these different collective behaviors arise from the physical interactions among migrating cells, and how to connect the known individual behaviors of cells to their collective behavior in groups of different cell numbers.

During cell-cell contact, individual cells show very characteristic behaviors. Studies on the kinematics and physical interactions between two colliding cells have revealed that cells retract their lamellipodium upon frontal contact with another cell, a phenomenon known as *Contact Inhibition of Locomotion* (CIL)^2, 10–12^. Studies of CIL have shown that cell pairs display an effective repulsion upon collision^11–14^ that is at odds with known coherent collective behavior of groups of cells both *in vitro* and *in vivo* ^1, 3^. Recent experiments have shown that cells can display both CIL in collisions between cell pairs and the formation of coherently moving cells when in larger groups^13^, suggesting that the same underlying mechanism of physical interaction between ceslls can give rise to both behaviors. In addition to CIL, recent *in vitro* studies indicate that cells repolarize away from pulling forces transmitted through cadherin-mediated cell adhesion and stabilize a lamellipodium in the opposite direction to the externally applied force^15, 16^. This *Force*-*Induced Repolarization* (FIR) establishes a mechanical feedback of cadherin-dependent adhesion forces from neighboring cells on the dynamics of cell polarization and traction forces. Both CIL and FIR play a major role in collective cell migration^11, 17–19^, as they couple cellular spatial configurations to the dynamics of cell traction forces via cell-cell contacts.

Most experimental studies concerning the physical aspects of collective cellular movements have focused on the migration of thousands of cells, such as in *in vitro* wound healing assays^20–23^. Accordingly, theoretical descriptions of these phenomena have been centered in the limit of very large numbers of cells, using both continuum theories^24, 25^ and discrete approaches based on self-propelled particles (SPP)^24, 26–29^. Continuum phenomenological descriptions have provided important insights into the generic behaviors of collective cellular movements at length scales much larger than cell size^24, 25^. Discrete SPP models inspired by flocking or schooling behavior of animal groups can reproduce coherent collective cell behavior through local velocity alignment rules^24, 29^. These models have been shown to successfully reproduce important features of large scale collective cell behavior, but do not explain important features of the dynamics of small groups of cells in which the specific characteristics of cellular interactions, including behaviors such as CIL or FIR, may play an important role. In general, SPP models can be used to describe the dynamics of small groups of cells and study the effects of important cell behaviors and parameters. Indeed, models of SPP have started to explore the role of CIL in the collective dynamics of cells in 2D, but either focus on large 2D monolayers or do not account for FIR^30–32^. It remains unclear how cell behaviors such as CIL and FIR contribute to collective cell migration, especially for small groups of cells, such as those observed in developing embryos or during cancer metastasis.

We introduce a theoretical description that successfully describes the motion of groups of cells of arbitrary numbers, from single cell motion to the collective migration of small groups of cells and large scale sheet migrations. The collective dynamics is obtained by balancing the forces in the system and specifying the dynamics of traction forces (or cell polarization) for individual cells, accounting for both CIL and FIR. We show that small groups of cells (3 or more cells) display coherent collective behavior, with persistence times that depend on the group size, despite their effective repulsion during the collision of cells pairs. We find an optimal size for small groups of cells that depends on cellular adhesion and traction strengths and maximizes the persistence of their coherent motion. Beyond small groups of cells, our description reproduces the diffusive behavior of individual cells in the absence of external cues, the observed behaviors upon pairwise cell collisions, as well as the traction force profiles reported in large scale cell migrations. Finally, we show that groups of identical cells can display coherent collective behavior or dispersal behavior by changing their confinement.

## Theoretical Description

**Figure 1:**
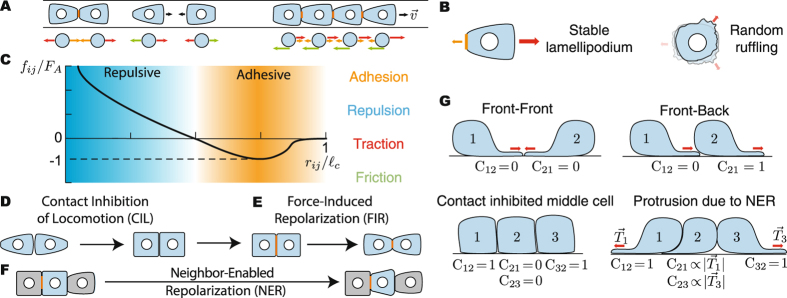
Description of the system, interaction forces and phenomenological cell behaviors. (**A**) Schematic representation of cells moving along a 1D strip (top) and particle-based representation of the system (bottom). Cells can be subject to adhesion forces (orange), excluded volume repulsion forces (blue) and friction forces (green), as well as generate traction forces (red). (**B**) Schematic representation of lamellipodial ruffluing (right) and a stable lamellipodium (left). (**C**) Pairwise interaction forces *f*_*ij*_ between cells as a function of their relative distance. Schematic representation of CIL (**D**) and FIR (**E**), leading to an effective repulsion between cells. (**F**) Schematic representation of neighbor-enabled repolarization (NER). (**G**) Schematic representation of cellular configurations during collisions and the associated values of the contact matrix *C*_*ij*_ for each configuration and cell.

We seek a minimal theoretical description accounting for key phenomenological observations regarding cell-cell interactions, namely CIL and FIR. To this end, we describe cells as particles and consider the pairwise physical interactions between them when moving along a 1D strip (Fig. 1A). While minimal, the 1D geometry has proven very useful to study collective cell migration at the experimental level^13, 14, 33^, as it simplifies the system considerably while preserving the essential features of collective cell migration.

### Particle-based description of single cell movements

In order to control their movements, cells regulate the forces they apply on their surroundings. A given cell generates a traction force $\vec{T}$ that causes its movement. Both dissipative processes inside the cell and friction with the substrate lead to a friction force opposing the cell movement which, in its most basic form, reads $-\xi \vec{v}$, with *ξ* being an effective friction coefficient and $\vec{v}$ the cell velocity. For the specific case of a single cell, it is instructive to consider also the effect of an external force ${\vec{F}}_{ext}$, as previously done experimentally by applying a controlled force with optical or magnetic tweezers^15, 16^. Neglecting inertial terms, force balance on the cell reads

1 $$\xi \vec{v}=\vec{T}+{\vec{F}}_{ext},$$

and specifies the cell velocity $\vec{v}$ that results from the forces in the system. While the external force in Eq. 1 is given and fixed, traction forces are generated by lamellipodial protusions and therefore controlled by their dynamics. The direction of lamellipodial extension, and consequently the direction of the traction force $\vec{T}$, depends on the direction of cell polarization as dictated by the intracellular localization of polarization factors such as RhoA, Cdc42 and Rac^9, 34^. In the absence of instructive external cues (biochemical or mechanical), cells constantly produce lamellipodial ruffles in random directions^34–36^ that decay over a timescale *τ*_*T*_ (protrusion lifetime), which characterizes the persistence of traction along a specific spatial direction (Fig. 1B). The timescale *τ*_*T*_ accounts here for the time necessary to repolarize the cell at a molecular level (i.e., changing the molecular polarity of the cell) and physically (rebuilding the lamellipodium), and is therefore associated with the cell (traction) repolarization time. Accounting for FIR due to an externally applied force^15, 16^, the dynamics of traction forces can be written as

2 $${\tau }_{T}\frac{d\vec{T}}{dt}=-\vec{T}-{T}_{M}{\hat{F}}_{ext}+{T}_{R}\,\hat{\eta },$$

where ${\hat{F}}_{ext}\equiv {\vec{F}}_{ext}/|{\vec{F}}_{ext}|$ is the direction of the applied external force, *T*_*R*_ is the characteristic force scale of individual lamellipodial ruffling and $\hat{\eta }$ is a random unit vector, denoting a delta-correlated white noise with unit variance, namely $\langle {\eta }_{i}(t){\eta }_{j}(t^{\prime} )\rangle ={\delta }_{ij}\delta (t-t^{\prime} )$. The force scale *T*_*M*_ represents the maximal force that a lamellipodium stabilized by the presence of an external cue can generate (Fig. 1B). While we do not consider the effect of external biochemical cues in this study, including them is straightforward.

### Systems with multiple cells

In a system with *N* cells (from *N* = 2 to *N* → ∞), cells apply forces on each other that affect their dynamics at different levels. Considering the forces that cells apply on each other, force balance on cell *i* reads

3 $$\xi {\vec{v}}_{i}={\vec{T}}_{i}+\sum _{j\ne i}\,{\vec{f}}_{ji},$$

where ${\vec{f}}_{ji}={F}_{A}\,f({r}_{ji}){\hat{r}}_{ji}$ is the force that cell *j* applies on cell *i*. From the perspective of cell *i*, ${\vec{f}}_{ji}$ is thus an external force along the direction ${\hat{r}}_{ji}=({\vec{r}}_{j}-{\vec{r}}_{i})/(|{\vec{r}}_{j}-{\vec{r}}_{i}|)$, where ${\vec{r}}_{i}$ and ${\vec{r}}_{j}$ are the cells’ positions. In contrast to the constant external force considered above for the one cell case, the magnitude of the intracellular forces changes with the cells’ configuration and we assume it depends only on the distance ${r}_{ji}=|{\vec{r}}_{j}-{\vec{r}}_{i}|$ between the cells. More specifically, it is characterized by a repulsive region, accounting for volume exclusion, and an attractive part, accounting for cell adhesion, with a attractive force *F*_*A*_ specifying the adhesion strength of the contact between two cells (Fig. 1C and Methods). We account for the finite size of the cell ${\ell }_{c}$ by setting a cutoff in the pairwise interaction force between cells at ${r}_{ji}={\ell }_{c}$ (*f*(*r*) = 0 if $r > {\ell }_{c}$) that prevents cell interactions if separated by more than the cell size (Methods).

Beyond the direct effect that forces from neighboring cells have on the motion of a given cell (Eq. 3), these forces also act as cues for cell repolarization and, as a consequence, affect the dynamics of the traction force exerted by the cell. As described above for a single cell, we account for FIR in the dynamics of traction forces, namely

4 $${\tau }_{T}\frac{d{\vec{T}}_{i}}{dt}=-{\vec{T}}_{i}-{T}_{M}\,\sum _{j\ne i}\,{{\rm{C}}}_{ij}\,{\rm{\Theta }}\,[f({r}_{ji})]\,{\hat{r}}_{ji}+{T}_{R}\,{\hat{\eta }}_{i},$$

where Θ(·) is the Heaviside function and allows only pulling forces to cause FIR. In addition to FIR, it is necessary to account for the effect of CIL (and other contact or exclusion effects) on traction forces when cells come into contact (Fig. 1D–F). We phenomenologically account for these processes using a contact matrix C_*ij*_ which we describe in details below.

When two cells collide, the observed lamellipodial retraction characteristic of CIL can be mathematically accounted for by expressing the contact matrix C_*ij*_ as ${{\rm{C}}}_{ij}=\mathrm{(1}-{\hat{r}}_{ji}\cdot {\hat{T}}_{i})/2$ (Fig. 1G). In 1D, C_*ij*_ is simply a Boolean matrix with zero values for configurations in which the lamellipodium frontally contacts the other cell, leading to lamellipodial retraction, and a value of one otherwise, allowing the formation of the lamellipodium (Fig. 1D,G and Eq. 4). Importantly, while we phenomenologically account for the observed retraction of the lamellipodium upon collision, we do not impose a repolarization of the lamelipodium away from the contact. We find that this repolarization, commonly associated with CIL^2, 11^, occurs naturally within our description as a consequence of FIR (Fig. 1D,E), which causes tractions to repolarize away from pulling forces established between cells upon collision, as suggested in recent experiments^17, 18^.

When *N* > 2, some cells may be contacted on all sides by other cells (Fig. 1A,E) and, according to CIL, these cells would not be able to generate any stable lamellipodium. However, experimental data from 1D cell clusters and 2D wound healing experiments suggests that cells contacted on all sides can generate cryptic (stable) lamellipodia^13, 37, 38^. In wound healing experiments, cells just behind the wound edge (second cell layer) generate stable lamellipodia in the same direction as that of already polarized cells at the leading edge. 3D imaging of cells in such expanding monolayers suggests that upon polarization, cells undergo shape changes that open spaces at their rear end, enabling neighboring trailing cells to protude lamellipodia^38^. This effect, which we call *Neighbor*-*Enabled Repolarization* (NER), does not specify the direction of cell repolarization. It instead permits a cell *i* to protude a cryptic lamellipodium if the neighboring cell *j* is polarized away from cell *i* (Fig. 1E,G). While NER can be simply due to shape changes upon cell polarization, other biochemical mechanisms can effectively generate the same effect, as recently proposed^18, 37, 39^. We mathematically account for NER and CIL in the contact matrix C_*ij*_ (Fig. 1G) which, for 1D systems with arbitrary number of cells, can be written as

5 $${{\rm{C}}}_{ij}=\frac{1-{\hat{r}}_{ji}\cdot {\hat{T}}_{i}}{2}\,[1+\sum _{k\ne j,k\ne i}\,[(\frac{1-{\hat{r}}_{ji}\cdot {\hat{T}}_{k}}{2})\,\frac{|{\vec{T}}_{k}|}{{T}_{M}}-1]].$$

The movement of each cell in a system with *N* cells is governed by Eqs 3, 4 and 5. Combining these equations and normalizing lengths with the cell size ${\ell }_{c}$, forces with the adhesion force scale *F*_*A*_, and time with the timescale ${\tau }_{M}=\xi {\ell }_{c}/\sigma$ associated with mechanical relaxation, we obtain three dimensionless parameters that control the dynamical regimes of the system, namely *T*_*M*_/*F*_*A*_, *T*_*R*_/*F*_*A*_ and *τ*_*T*_/*τ*_*M*_. The parameters *T*_*M*_/*F*_*A*_ and *T*_*R*_/*F*_*A*_ compare the relative strengths of traction forces generated by stable lamellipodia and lamellipodial ruffling to adhesion forces, respectively. Finally, the ratio *τ*_*T*_/*τ*_*M*_ compares the repolarization timescale *τ*_*T*_ to the time scale *τ*_*M*_ that a cell requires to reach mechanical equilibrium.

## Results

We study the cellular movements in systems of *N* cells by numerically solving Eqs 3, 4 and 5 (Methods), as analytical solutions are difficult to obtain due to the highly non-linear nature of the dynamics.

### Single cell movements

In the absence of any external cues (${\vec{F}}_{ext}=0$), the cellular movements resulting from integrating Eqs 1 and 2 are ballistic at short time scales (*t* < *τ*_*T*_), with average velocity *T*_*R*_/*ξ*, and diffusive at timescales longer than the traction persistence time scale *τ*_*T*_, with diffusion constant *D* given by $D={T}_{R}^{2}{\tau }_{T}/{\xi }^{2}$. The timescale of velocity autocorrelation decay is *τ*_*T*_, indicating that *τ*_*T*_ is indeed the persistence timescale of cellular motion. In the presence of an external pulling force (mechanical cue; ${\vec{F}}_{ext}\ne 0$), the cell polarizes away from the pulling force and, at time scales longer than *τ*_*T*_, it generates a traction force $-{T}_{M}\,{\hat{F}}_{ext}$ opposing the external force (Eq. 2). Force balance (Eq. 1) shows that the average velocity of the cell is $\vec{v}=-({T}_{M}/\xi )\,(1-|{\vec{F}}_{ext}|/{T}_{M})\,{\hat{F}}_{ext}$, indicating that the cell moves away from the pulling force at a speed that decreases linearly with the applied pulling force, with a maximal velocity *T*_*M*_/*ξ* and a stall force *T*_*M*_, analogous to molecular motors^40^.

### Collisions between two cells (N = 2)

**Figure 2:**
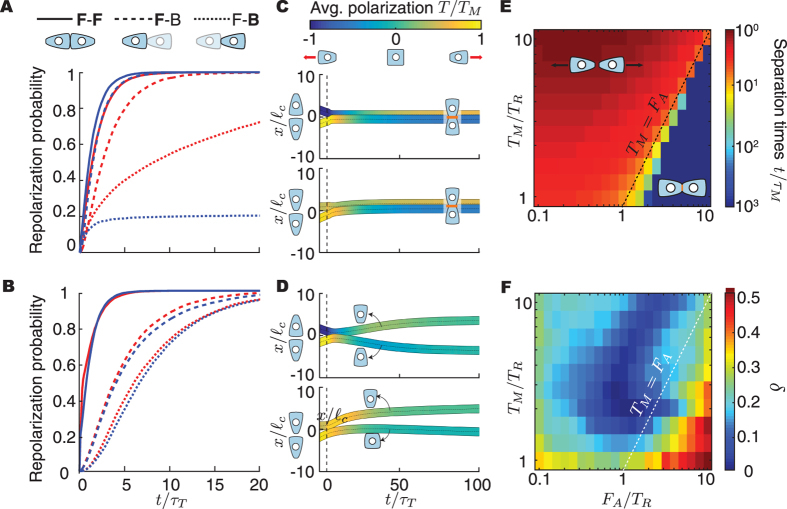
Collision dynamics between two cells. Cell repolarization probabilities (**A**,**B**) and average trajectories (**C**,**D**) of colliding cells after F-F (triangles) and F-B collisions for both trailing (**F**-B, squares) and leading (F-**B**, circles) cells, and high ((**A**,**C**) *T*_*M*_/*T*_*R*_ = 1, *F*_*A*_/*T*_*R*_ = 10) and low ((**B**,**D**) *T*_*M*_/*T*_*R*_ = 10, *F*_*A*_/*T*_*R*_ = 1) adhesion levels. Red and blue in (A,C) lines correpsond to *τ*_*T*_/*τ*_*M*_ = 1 and *τ*_*T*_/*τ*_*M*_ = 10 respectively. Color code in (**C**,**D**) shows ensemble average of cell polarization during collision. Width of trajectory represents cell size ${\ell }_{c}$. *τ*_*T*_/*τ*_*M*_ = 1 in panels (C,D). (**E**) Cell separation time (normalized to *τ*_*M*_) for the different parameters in the problem. Cell separation times increase sharply, indicating that cells essentially remain attached, when *F*_*A*_ > *T*_*M*_. (**F**) Comparison of theoretical predictions to experimental data in ref. 13. The measured discrepancy *δ* (Methods) between the experimental data and the theoretical predictions is shown (color coded) for varying values of *T*_*M*_/*T*_*R*_ and *F*_*A*_/*T*_*R*_. Minimal values of discrepancy were found for ${T}_{M}/{T}_{R}\simeq 2-3$ and ${F}_{A}/{T}_{R}\simeq 0.8-1.1$. *τ*_*T*_/*τ*_*M*_ = 1 for both panels (E,F).

Most cell-cell collision experiments measure the repolarization probabilities of two colliding cells at a fixed time after collision and for all possible initial cell-cell configurations before collision, namely front-front (F-F) and front-back (F-B) collisions^13, 14^ (Fig. 2). Simulations of cell collisions indicate that cell repolarization is always faster in F-F collisions for any value of the different parameters in the system (Fig. 2A,B). In F-B collisions, the trailing cell (**F**-B) engages in a frontal collision with the leading cell (F-**B**), which is contacted at its back end, and always repolarizes faster than the leading cell, as observed experimentally^13^. When the force of adhesion is larger than the forces produced by stable lamellipodia (*F*_*A*_ > *T*_*M*_), cells remain attached to each other after collision (Fig. 2E), with traction forces oriented away from each other (Fig. 2A,C). In contrast, when traction forces are larger than adhesion forces (*T*_*M*_ > *F*_*A*_), cells separate shortly after collision and move away from each other (Fig. 2B,D), with separation times being similar to the repolarization time scale *τ*_*T*_ (Fig. 2E). These configuration-dependent behaviors arise from the combined action of CIL and FIR, which depend on the mechanical state of each cell configuration.

Comparing published experimental data of repolarization probabilities in 1D collisions between NRK-52E cell pairs^13^ to our theoretical predictions (Fig. 2F and Methods), we find that the minimal discrepancy is obtained for ${T}_{M}/{T}_{R}\simeq 2-3$ and ${T}_{A}/{T}_{R}\simeq 0.8-1.1$, indicating that NRK-52E cells generate stable traction forces *T*_*M*_ two to three times larger than adhesion forces *F*_*A*_ and that ruffling forces (*T*_*R*_) alone are strong enough to separate the cells (${T}_{A}/{T}_{R}\simeq 0.8-1.1$).

### Small groups of cells (2 < N ~ 10)

**Figure 3:**
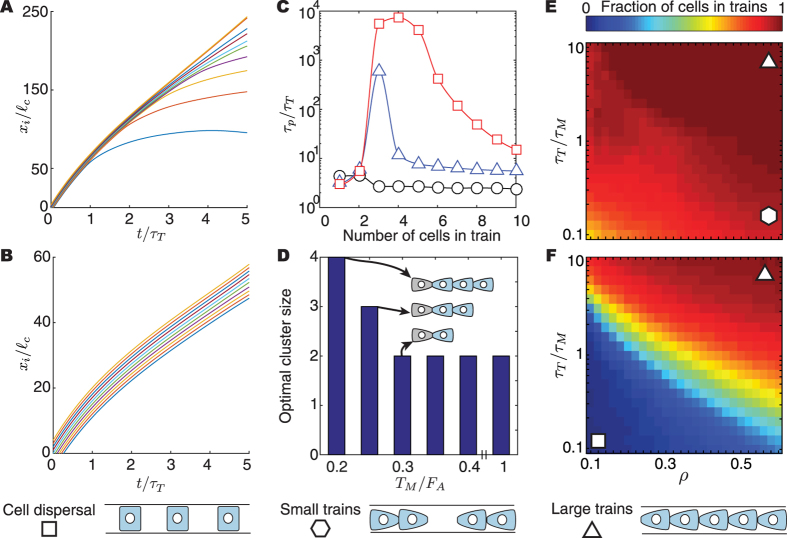
Persistence and dynamics of cell trains. (**A**,**B**) Position kymograph of 10-cell trains for low ((**A**) *T*_*M*_/*F*_*A*_ = 0.2) and high ((**B**) *T*_*M*_/*F*_*A*_ = 0.8) adhesion levels. (**C**) Average persistence time of trains for varying adhesion levels: *T*_*M*_/*F*_*A*_ = 0.2, 0.25, 0.8 (red, blue and black, respectively). (**D**) Optimal train (cluster) size as a function of *T*_*M*_/*F*_*A*_. *τ*_*T*_/*τ*_*M*_ = 10 and *F*_*A*_/*T*_*R*_ = 10 in panels (A–D). (**E**,**F**) Dynamics of train formation as a function of density (confinement) and *τ*_*T*_/*τ*_*M*_ for high ((**E**) *T*_*M*_/*F*_*A*_ = 0.5, *F*_*A*_/*T*_*R*_ = 10) and low ((**F**) *T*_*M*_/*F*_*A*_ = 2, *F*_*A*_/*T*_*R*_ = 10) adhesion levels.

To characterize the collective behavior of small groups of cells (or cell trains), we first simulate compact groups of identically polarized cells and study their persistence. When traction forces are larger than adhesive forces (*T*_*M*_ > *F*_*A*_), the initially coherent train starts losing its persistence over a timescale *τ*_*T*_, with cells at the trailing end repolarizing and detaching from the train (Fig. 3A). In contrast, when cell-cell adhesion is larger than traction (*F*_*A*_ > *T*_*M*_), coherent cell trains with persistent average cell polarization exist (Fig. 3B) over timescales that depend on the number of cells in the train (Fig. 3C). We observe an optimal train size for each ratio *T*_*M*_/*F*_*A*_ that maximizes the persistence time *τ*_*p*_ of the train, which can become orders of magnitude larger than *τ*_*T*_ (Fig. 3C). This optimal train size increases for increasing adhesion strength relative to the cell traction forces (Fig. 3D). Despite the existence of CIL, persistent trains with coherent polarization can exist because of NER. Importantly, the trailing cell in the train always repolarizes away from the average train polarization because of CIL and is dragged forward by the collective train motion (Fig. 3D), as observed experimentally^13^.

Beyond small groups of fixed number of cells, we study the collective behavior of cells moving along a 1D strip with periodic boundary conditions (ring geometry). In this case, the behavior of the system depends on the average cell density $\rho \equiv N{\ell }_{c}/L$ (with *L* being the perimeter of the ring), which parameterizes cellular confinement. If the adhesion strength between cells is much larger than the traction forces exerted by stably polarized cells (*F*_*A*_ > *T*_*M*_), then either one or several groups of cells that move coherently dominate the system for almost any value of initial density (Fig. 3E). In contrast, for small values of cell adhesion strength (*F*_*A*_ < *T*_*M*_), we find cell dispersal behavior at low densities, with cells covering the entire length of the track and maximizing their average distance from each other (Fig. 3F), a result that could explain cell dispersal behaviors observed *in vivo* ^41, 42^. Even in these low adhesion conditions, cells can form coherent trains at large densities. These trains are dynamic structures, with cells being added and removed from the train, but keeping a finite size. This is because at large densities the typical time scale of adding a new cell to a train can be shorter than the time scale *τ*_*T*_ for cells to repolarize and separate from the train. The transition between dispersal and coherent train formation occurs by solely changing the cell density, even if no cell parameters (traction, adhesion, polarization time, etc.) are changed. This indicates that a given cell type can display both dispersal behavior and coherent train formation at different densities (confinement conditions), as suggested in recent experiments^43^.

### Large cell colonies (N $\gg$ 10)

**Figure 4:**
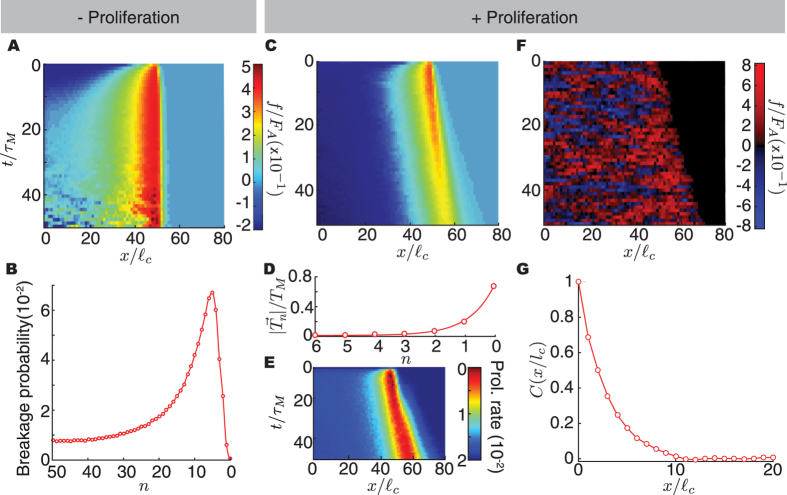
Expansion of large cell colonies. (**A**,**C**) Ensemble average intercellular force kymographs of expanding cell colonies in the absence (**A**) and presence (**C**) of cell proliferation. (**B**) Probability of colony breakage in the absence of cell proliferation as a function of the distance from the edge of the colony. (**D**) Spatial profile of traction forces from the edge of the colony in the presence of cell proliferation. (**E**) Ensemble average cell proliferation kymograph showing spatiotemporal variations during colony expansion. (**F**) Intercellular force kymograph from a single simulation run. (**G**) Ensemble spatial autocorrelation of intercellular force which can be fitted to an exponential with a characteristic length of 2.8*l*_*c*_. In all cases the value of *T*_*M*_/*F*_*A*_ = 0.25, *F*_*A*_/*T*_*R*_ = 10 and *τ*_*T*_/*τ*_*M*_ = 0.1.

We study large colonies of strongly adhesive cells (*F*_*A*_ > *T*_*M*_) in 1D, as this situation mirrors sheet migration in 2D wound healing experiments. Cells are initialized in a configuration where they are attached to each other and have random polarizations. In all cases, cells at the edge develop polarizations away from the colony and start pulling on it. A polarization wave that propagates from the edge to the interior of the cell colony transfers the forces generated at the edge to cells deep in the colony (Fig. 4A). If the buildup of intercellular forces within the colony exceeds the maximal adhesion force between cells, the colony breaks, with the highest probability of breakage occurring where the intercellular forces are maximal on average (Fig. 4B). The possibility of colony breakage occurs because cells inside the colony can develop cryptic lamellipodia, contributing to a collective buildup of forces that must be sustained by adhesion at cell-cell junctions. If cryptic lamellipodia did not exist, only cells at the edge would generate traction forces and this would not lead to sufficient forces at cell-cell junctions to cause colony breakage (for strongly adhering cells, i.e., *F*_*A*_ > *T*_*M*_). While both cryptic lamellipodia and the generation of traction forces inside the cell colony have been experimentally observed, colony breakage has not been reported. This can be for a number of reasons that we discuss in the *Discussion* section below.

All results above were obtained in the absence of cell proliferation. Since cell proliferation is present in most experiments on colony expansion^22, 44^, we study the role of cell proliferation in the propagation of intracellular forces within the colony. To this end, we simulate the dynamics of the colony as described above, but allowing cells to divide if the separation between them becomes larger than a critical length ${\ell }_{d}$ (the results described below do not qualitatively depend on the choice of ${\ell }_{d}$). We find that proliferation prevents the buildup of large intercellular forces deep in the colony, enabling it to continuously grow (Fig. 4C). This effect is equivalent to a fluidization of the cell colony at time scales larger than proliferation times^45^. Both the traction (Fig. 4D) and proliferation (Fig. 4E) spatial profiles decay over just a few cell sizes from the edge of the colony, as observed experimentally^22^. The penetration (decay) length scale of intercellular forces and proliferation are considerably larger than that of traction forces, in agreement with experimental observations^44^. Importantly, as previously observed in the expansion of 2D cell monolayers^22^, intercellular forces display large spatial heterogeneities (Fig. 4F). While these inhomogeneities are averaged out when performing ensemble averages over many simulations (Fig. 4A,C), they become apparent for single simulation runs (Fig. 4F). Calculation of the spatial autocorrelation function (Fig. 4G) and its associated autocorrelation length (Methods) indicate that the heterogeneities in intercellular forces span a few cell sizes, with the specific size of the inhomogeneities depending on the parameters of the system.

## Methods

### Particle-based simulations

Cells are simulated with an intercellular force consisting of a repulsive core up to ${\ell }_{c}/2$ and an attractive region between ${\ell }_{c}/2$ and ${\ell }_{c}$ (Fig. 1B). The exact functional form used is

6 $$f({r}_{ji})=\{\begin{array}{cc}c(\frac{{\ell }_{c}}{2{r}_{ji}}-\frac{{\ell }_{c}^{3}}{8{r}_{ij}^{3}}) & \text{when}\,0 < {r}_{ji} < 3{\ell }_{c}/4,\\ c(\frac{{\ell }_{c}}{2{r}_{ji}}-\frac{{\ell }_{c}^{3}}{8{r}_{ij}^{3}}){\rm{\Psi }}({r}_{ji}/{\ell }_{c}-3/4,1/4) & \text{when}\,3{\ell }_{c}/4\le {r}_{ji} < {\ell }_{c},\\ 0, & \text{when}\,{\ell }_{c}\le {r}_{ji},\end{array}$$

where

7 $${\rm{\Psi }}(x,a)=\{\begin{array}{cc}{e}^{-\frac{1}{1-(x/a-1{)}^{2}}} & \text{when}\,|x| < a,\\ 0 & \text{otherwise}\end{array}$$

is a bump function which is smooth at *a*, hence guaranteeing that *f*(*r*_*ji*_) is smooth when *r*_*ji*_ = *l*_*c*_. The constant *c* is chosen such that ${{\rm{\max }}}_{0 < {r}_{ji}\le {l}_{c}}\,f({r}_{ji})=1$. Eq. 3 is solved using an explicit Euler scheme, and the stochastic differential equation associated with the generation of traction, Eq. 4, is solved using the Euler-Maruyama method^46^. The timesteps of simulation were chosen adaptively based on the parameters of the system. All simulations were performed with custom computer codes.

### Simulations of collisions between two cells

Repolarization probabilities (Fig. 2A,B) were obtained by computing repolarization times in 25000 instances of collisions in a 1D box of length $100{\ell }_{c}$. The repolarization time is computed as the difference between the instant at which a cell is within ${\ell }_{c}$ distance of the other cell, and the instant at which it switches direction, as defined by a change in sign of its traction. To guarantee that repolarization was not transient, the direction of traction was tracked for a time *τ*_*T*_ after switching direction and only events in which the sign of traction did not revert were taken into account. Trajectories are mean displacements over 25000 instances of collisions, starting with tractions *T*_*M*_/2 for head-on collisions and tractions 3*T*_*M*_/4, *T*_*M*_/4 for rear-end collisions. Since we average many simulations for each set of parameters (ensemble average), error bars associated to simulation results are very small and not shown; simulation results are plotted as continuous or dashed lines (Fig. 2A–C).

The average separation times for each combination of *T*_*M*_/*T*_*R*_ and *F*_*A*_/*T*_*R*_ (Fig. 2E) were computed as the ensemble average (*N* = 10^4^) of the time required for two cells to separate. Cells were randomly initialized at a distance between 0.4*l*_*c*_ and 0.6*l*_*c*_ and the simulations were terminated after 10^4^ *τ*_*M*_ timesteps. If cells were still attached at that point, their separation time was set to 10^4^ *τ*_*M*_.

To calculate the difference between experimental data from ref. 13 and theoretical predictions (Fig. 2F), we first simulated 2500 instances of F-F, F-**B**, **F**-B collisions between two cells for different values of the parameters *T*_*M*_/*T*_*R*_ and *F*_*A*_/*T*_*R*_ (and fixed *τ*_*T*_/*τ*_*M*_ = 0.1) and obtain the cumulative repolarization probabilities ${P}_{F-F}^{sim}(t)$, ${P}_{{\bf{F}}-B}^{sim}(t)$ and ${P}_{F-{\bf{B}}}^{sim}(t)$ as a function of these parameters (using the same procedure as described above; Fig. 2A,B). The superscript *sim* refers to the fact that values were obtained from simulation. While in our simulations the cumulative repolarization probabilities depend on time, the experimental data from ref. 13 reports the repolarization probability 2 hours after cells collided. Since neither of the timescales *τ*_*T*_ or *τ*_*M*_ are explicitly known for NRK-52E cells^13^, the comparison of our simulation results to the experimental data requires comparing simultaneously at least two collision types, as for a single collision type it is always possible to find a time in the predicted cumulative repolarization probability that matches the experimental value of the repolarization probability. When comparing simulations and experimental data for two or more collision types simultaneously, there are enough constraints to make the comparison meaningful. For this reason, we compare simultaneously the three collision types (F-F, F-**B**, **F**-B) and define the measure of discrepancy between experimental and theoretical values, *δ*, as

8 $$\delta \,({F}_{A}/{T}_{R},{T}_{M}/{T}_{R})=\mathop{{\rm{\min }}}\limits_{t}\sqrt{\tfrac{1}{3}\,\{\tfrac{{({P}_{F-F}^{exp}-{P}_{F-F}^{sim}(t))}^{2}}{{({P}_{F-F}^{exp}+{P}_{F-F}^{sim}(t))}^{2}}+\tfrac{{({P}_{F-{\bf{B}}}^{exp}-{P}_{F-{\bf{B}}}^{sim}(t))}^{2}}{{({P}_{F-{\bf{B}}}^{exp}+{P}_{F-{\bf{B}}}^{sim}(t))}^{2}}+\tfrac{{({P}_{{\bf{F}}-B}^{exp}-{P}_{{\bf{F}}-B}^{sim}(t))}^{2}}{{({P}_{{\bf{F}}-B}^{exp}+{P}_{{\bf{F}}-B}^{sim}(t))}^{2}}\}},$$

where the values ${P}_{F-F}^{exp}$, ${P}_{F-{\bf{B}}}^{exp}$ and ${P}_{{\bf{F}}-B}^{exp}$ are the probabilities of repolarization 2 hours after collision, for each type of collision, reported in Desai *et al*.^13^, namely: ${P}_{F-F}^{exp}\simeq 0.87$, ${P}_{F-{\bf{B}}}^{exp}\simeq 0.18$ and ${P}_{{\bf{F}}-B}^{exp}\simeq 0.59$. The measure *δ* finds the time for which the discrepancy between theory and experiments is minimal and reports, for each value of the parameters *T*_*M*_/*T*_*R*_ and *F*_*A*_/*T*_*R*_, such discrepancy *δ*, which is shown in Fig. 2F.

### Simulations of groups of cells

To obtain position kymographs and train persistence times, the cells were initialized with identical tractions *T*_*M*_ spaced at a distance of $0.5{\ell }_{c}$. The persistence time *τ*_*p*_ corresponds to the time until either breakage or repolarization of the train occurs, i.e., when the distance between adjacent cells in the group becomes larger than ${\ell }_{c}$, or the time at which the cluster reverses direction, namely ${\sum }_{i}\,{\vec{T}}_{i} < 0$. The position kymographs and persistence times correspond to ensemble averages obtained from 10^4^ runs. Dynamic train formation is computed by simulating *N* cells in a 1-D box of length ${10}^{2}{\ell }_{c}$ with periodic boundary conditions (equivalent to a ring geometry). The fraction of cells in trains were determined as the cells existing in clusters of length greater than 2 cells as compared to the total number of cells. The system was simulated for times 10^3^ max {*τ*_*T*_,*τ*_*M*_} and was repeated for 10000 instances. Train fractions were obtained as ensemble averages of the ratio of the mean number of cells in trains to the total number of cells.

### Simulations of cell colonies

In all cases, 10^2^ cells were initialized in close proximity, with the distance between neighboring cells randomly chosen between $0.4{\ell }_{c}$ and $0.6{\ell }_{c}$. To quantify colony breakage, the cell position where the distance to neighboring cells exceeds ${\ell }_{c}$ is noted as the point of breakage. Breakage probabilities are breakage frequencies from 10^4^ runs. Cell division is modeled as the inclusion of a new cell at the midpoint of the segment joining the centers of two adjacent cells whose distance has exceeded $0.75{\ell }_{c}$. The newly formed cell starts with no traction. The parameter *T*_*M*_/*F*_*A*_ is chosen as 0.25 to mimic colony expansion in cell types with high adhesion (e.g., MDCK cells). Intercellular forces (Fig. 4A,C), breakage probabilities (Fig. 4B), traction profiles (Fig. 4D) and proliferation rates (Fig. 4E) correspond to ensemble averages over 10000 runs. The intercellular forces in the kymograph of Fig. 4F were obtained using a single simulation run of the same system.

The spatial autocorrelation function was calculated as

9 $$C(x/{\ell }_{c})=\frac{1}{N}\sum _{i=1}^{N}\frac{\sum _{t=1}^{{t}_{\text{max}}}({f}^{(i)}({x}_{0}/{\ell }_{c},t/{\tau }_{M})- < {f}^{(i)}({x}_{0}/{\ell }_{c}) > )({f}^{(i)}(|{x}_{0}+x|/{\ell }_{c},t/{\tau }_{M})- < {f}^{(i)}(|{x}_{0}+x|/{\ell }_{c}) > )}{\sum _{t=1}^{{t}_{\text{max}}}({f}^{(i)}({x}_{0}/{\ell }_{c},t/{\tau }_{M})- < {f}^{(i)}({x}_{0}/{\ell }_{c}) > {)}^{2}}\,,$$

where *f* ^(*i*)^ (*x*, *t*) refers to the intercellular force at position *x* at time *t* from the *i* ^th^ simulation run and 〈*f* ^(*i*)^ (*x*)〉 refers to the time average of the intercellular force at position *x* for the *i* ^th^ simulation run; *x*_0_ was chosen to be 25*l*_*c*_ from the middle of the colony; *t*_max_, which is the maximum simulation time, was chosen to be 500*τ*_*M*_; and *N* = 100 is the number of ensembles over which the autocorrelation function was computed. The exact position of *x*_0_ in the colony does not affect the value of the autocorrelation function obtained.

## Discussion

We presented a theoretical description of cell migration that accounts for known individual cell behaviors, such as CIL and FIR, and is able to reproduce the motion of a single cell, two cell collisions, small groups of cells and large colonies. This description provides a unified framework to connect the large number of experiments in different conditions and with different cell types. Moreover, it allows a direct connection between specific molecular perturbations in cell adhesion, cell polarization, the generation of traction forces and mechanical feedback, and their effect on collective cell migration.

At the single cell level, our predictions of diffusive movements at time scales longer than the traction persistence time, are in good agreement with experimental observations showing diffusive cell movements at long timescales^47, 48^. Our results predict that the diffusion constant of cellular movements depends quadratically with the cell’s traction force. This prediction could be experimentally tested by measuring the magnitude of traction forces using traction force microscopy while monitoring cellular movements. In addition, the predicted dependence of the cell velocity on an applied external force can potentially be measured using magnetic tweezers in a similar way as in previous experiments^16^.

Beyond single cell movements, the observed behaviors in collision experiments on CIL^13, 14, 17^ arise naturally in our description if both CIL and FIR are taken into account. Importantly, in the theoretical description presented above, CIL involves only lamellipodial retraction but does not impose repolarization away from contact; repolarization is a consequence of the pulling forces acting on the cell via FIR. While it is typically assumed that CIL involves repolarization, our description highlights the importance of considering the separate effects of lamellipodial retraction and force-dependent repolarization, as suggested by recent experimental results^10, 17, 18^. Indeed, some cell types show lamellipodial retraction upon contact, but no repolarization^10, 12, 49^. Our predictions indicate that the dynamics of repolarization, characterized by the cumulative probability of repolarization (Fig. 2), are very different for distinct collision types and depend strongly on parameters such as adhesion strength or traction force (*T*_*M*_/*F*_*A*_) as well as the traction repolarization time and the mechanical relaxation time (*τ*_*T*_/*τ*_*M*_). These parameters can be experimentally varied using drugs targeting force generation or cell adhesion, and the dynamics of cell polarization could be monitored with polarization markers. Having a quantitative understanding of the behavior of cells during collisions would considerably help understand their behavior in larger groups.

Several experimental works have shown that coherently moving cell groups emerge even for cells types that display repulsion upon collision^2, 13^. Our theoretical predictions indicate that this phenomenon can be explained by a stabilization of lamellipodia enabled by neighboring polarized cells (NER) through either physical or biochemical mechanisms. In the absence of NER, our analysis predicts that no coherently moving cell groups can exist, as CIL prevents their formation. Since NER is directly related to the existence of cryptic lamellipodia, experiments exploring the physical and biochemical cues enabling cells to generate lamellipodia when contacted on all sides may help understand their collective behavior. In particular, experiments to characterize how polarization of cells affects the ability of their neighbors to polarize and generate cryptic lamellipodia may help understand the role of NER.

We find an optimal group number that maximizes migration persistence of small groups of cells (Fig. 3C,D), which could explain why collective migration of small cell groups is often observed in developing embryos^1, 3, 4^ and cancer metastasis^6^. This prediction can directly be tested in 1D systems by measuring either switches in the direction of group motion or group breakage for groups of cells of different numbers (no cell proliferation) and for different cell adhesion strength. Our results also indicate that by varying the cell density alone (or confinement), with no changes in cell specific parameters (for a given cell type), both coherently moving cell trains or cell dispersal behavior can be observed (Fig. 3C,F). These predictions suggest that several experimentally observed behaviors^41–43^, such as cell dispersal and coordinated group migration, can be achieved by varying cellular confinement. We also find that, in addition to cell density, cell specific parameters can control the ability of cells to form coherently moving groups or disperse (Fig. 3C,F). Experiments to test these results could be realized in 1D systems by controlling the cell seeding density and monitoring cellular movements in the absence of cell proliferation.

Beyond small groups, we find that in the absence of cell proliferation, large cell colonies may break up into smaller groups as a consequence of large intercellular forces that build up within the colony. However, colony breakage has not been observed in 2D cell monolayers, even in the absence of cell proliferation. Since colony breakage occurs in our simulations only if intercellular pulling forces become larger than the cell-cell adhesion strength, it is likely that the cells used in many of these experiments (e.g., MDCK cells^22, 44^) adhere so strongly to each other that breakage is never observed. Our predictions indicate that lowering mildly the adhesion strength between cells should enable portions of the colony located close to the migrating edge (where intercellular forces are predicted to be largest) to break off. Another possibility is that colony breakage is an effect observed only in 1D geometries, as in 2D cell monolayers, the larger number of neighbors per cell may be able to sustain the forces that build up within the monolayer and help prevent breakage. Our results show that, at least in 1D, the presence of cell proliferation can help avoid colony breakup by hindering the buildup of large intercellular forces. While the 1D system studied here is not equivalent to a 2D cell monolayer, the predicted profile of cell proliferation consistent with previous experimental observations^44^.

The theoretical description presented here shows that the collective migration of small groups of cells can be understood within the same framework as single cell migration and the expansion of large colonies. Extensions of this work to 2D and 3D systems^50^, as well as the consideration of cell shapes or biochemical signaling, will help elucidate how the different modes of collective migration emerge in developing embryos.
